# Outcomes of treatment cessation after switching to subcutaneous vedolizumab treatment in inflammatory bowel diseases

**DOI:** 10.1177/17562848241290636

**Published:** 2024-10-16

**Authors:** Péter Bacsur, Tamás Resál, Patrícia Sarlós, Ákos Iliás, Liza Dalma Sümegi, Diána Kata, Anett Dávid, Bernadett Farkas, Emese Ivány, Anita Bálint, Zsófia Bősze, Anna Fábián, Renáta Bor, Zoltán Szepes, Waqqas Afif, Talat Bessissow, Klaudia Farkas, Péter L. Lakatos, Tamás Molnár

**Affiliations:** Department of Medicine, Albert Szent-Györgyi Medical School, University of Szeged, Szeged, Hungary; HCEMM-USZ Translational Colorectal Research Group, Szeged, Hungary; Department of Medicine, Albert Szent-Györgyi Medical School, University of Szeged, Szeged, Hungary; Division of Gastroenterology, First Department of Medicine, Medical School, University of Pécs, Pécs, Hungary; Department of Internal Medicine and Oncology, Semmelweis University, Budapest, Hungary; Department of Internal Medicine and Oncology, Semmelweis University, Budapest, Hungary; Institute of Laboratory Medicine, Albert Szent-Györgyi Medical School, University of Szeged, Szeged, Hungary; Department of Medicine, Albert Szent-Györgyi Medical School, University of Szeged, Szeged, Hungary; Department of Medicine, Albert Szent-Györgyi Medical School, University of Szeged, Szeged, Hungary; Department of Medicine, Albert Szent-Györgyi Medical School, University of Szeged, Szeged, Hungary; Department of Medicine, Albert Szent-Györgyi Medical School, University of Szeged, Szeged, Hungary; Department of Medicine, Albert Szent-Györgyi Medical School, University of Szeged, Szeged, Hungary; Department of Medicine, Albert Szent-Györgyi Medical School, University of Szeged, Szeged, Hungary; Department of Medicine, Albert Szent-Györgyi Medical School, University of Szeged, Szeged, Hungary; Department of Medicine, Albert Szent-Györgyi Medical School, University of Szeged, Szeged, Hungary; Division of Gastroenterology, McGill University Health Center, Montreal, QC, Canada; Division of Gastroenterology, McGill University Health Center, Montreal, QC, Canada; Department of Medicine, Albert Szent-Györgyi Medical School, University of Szeged, Kálvária Avenue 57, Szeged H-6725, Hungary; HCEMM-USZ Translational Colorectal Research Group, Szeged, H-6728, Hungary; Division of Gastroenterology, McGill University Health Centre, Montreal General Hospital, 1650 Avenue Cedar, D7-201, Montreal, QC H3G 1A4, Canada; Department of Internal Medicine and Oncology, Semmelweis University, Koranyi S 2A, Budapest H-1083, Hungary; Department of Medicine, Albert Szent-Györgyi Medical School, University of Szeged, Kálvária Avenue 57, Szeged H-6725, Hungary

**Keywords:** Crohn’s disease, IBD, patient experience, subcutaneous vedolizumab, switching, ulcerative colitis

## Abstract

**Background:**

The usability of subcutaneous vedolizumab (s.c. VDZ) treatment in inflammatory bowel diseases (IBD; ulcerative colitis (UC), Crohn’s disease (CD)) has been proven via clinical trials while real-world data collection is ongoing.

**Objectives:**

Our study evaluates the effectiveness, safety, patients’ preferences, and psychological factors associated with s.c. VDZ treatment, after switching from intravenous (i.v.) formulation.

**Design:**

Prospective, multicenter cohort study including IBD patients switching from i.v. VDZ to s.c. treatment and were evaluated over 52 weeks.

**Methods:**

Serum VDZ levels and C-reactive protein (CRP) were measured at the baseline and w52. At w12, a questionnaire on the patient’s satisfaction and psychological characteristics was administered. The primary outcome was the drug persistence rate (cessation was due to loss of response (LOR), adverse events, patient request, and other causes) at w52, while the secondary outcomes were the changes in the clinical corticosteroid-free remission (CSFR) and biochemical remission (BR; CRP ⩽ 5 mg/L) rates, safety issues, serum drug levels, patients’ preferences, and psychological features.

**Results:**

In total, 70 IBD patients were evaluated (32 CD patients, 38 UC patients; male/female ratio: 41.4%; median age: 43.2 years). In the CD group, 81.3% were in CSFR and 65.6% were in BR, while in the UC group, 71.7% were in CSFR and 69.4% were in BR. Overall, 17.1% of the patients ceased s.c. VDZ treatment after a median of 26.2 (interquartile range 20–47) weeks. LOR was registered in 3/12 ceased patients. In addition, CSFR and BR rates were stable, while serum VDZ levels increased by w52 (*p* < 0.001).

**Conclusion:**

The transition from i.v. to s.c. VDZ treatment was effective, the overall persistence rate was associated with high serum drug levels, and no novel safety issues were reported. Although s.c. administration after induction can save resources, some patients still insisted on i.v. VDZ treatment, due to its proven formulation.

## Introduction

Biological treatments have revolutionized the treatment of inflammatory bowel diseases (IBD; Crohn’s disease (CD) and ulcerative colitis (UC)),^1–3^ with the potential to achieve mucosal healing or transmural healing based on the treat-to-target approach proposed by STRIDE-II consensus.^4^ Specifically, vedolizumab (VDZ) is a humanized IgG1 monoclonal antibody that targets α4β7 integrin and inhibits the interaction with mucosal addressin cell adhesion molecule-1, resulting in the prevention of leukocyte extravasation to the inflamed tissue. Meanwhile, the efficacy and safety of VDZ in IBD have been reported in related research.^5,6^ The development of biological agents has also resulted in subcutaneous (s.c.) admission since adalimumab and ustekinumab maintenance treatment is performed by s.c.^7–10^ Meanwhile, real-world verification of s.c. infliximab (compared to intravenous (i.v.) formulation) in IBD is ongoing.^11^

Clinical trials have verified the usability of s.c. VDZ, as a maintenance treatment in IBD after i.v. induction.^12,13^ Due to the injection form, elevated serum VDZ levels were detected, which may be due to the limited bioavailability, slow absorption rate, and lower peak concentrations.^14^ The exposure–efficacy relationship for VDZ has raised issues, which were demonstrated by observational trials; however, real-world experiences are contradicting in the case of switching to s.c. formulation in a patient who lost effectiveness on i.v. VDZ.^15^

Although real-world data of switching from i.v. to s.c. administration is ongoing, several papers have collected nationwide experiences, including results within a year.^16–25^ Meanwhile, trials assessing patients’ preferences, satisfaction, and psychological components of decision-making are almost lacking.^17,21^

Therefore, our study aimed to evaluate the real-world effectiveness and safety of s.c. VDZ maintenance treatment after switching from i.v. formulation and focuses on the patient’s experiences and satisfaction after switching. We also aimed to assess the psychological components of decision-making resulting from the failure of the self-injection treatment.

## Materials and methods

### Study design and participants

This prospective, multicenter cohort study focuses on four tertiary IBD centers in Hungary (Department of Medicine, University of Szeged, Szeged; First Department of Medicine, University of Pécs, Pécs; and First Department of Medicine, Semmelweis University, Budapest) and Canada (McGill University Health Center, Montreal, Quebec). Consecutive IBD patients with ⩾18 years on i.v. VDZ maintenance treatment who agreed to switch to s.c. VDZ formulations were enrolled between May 1, 2021 and July 31, 2022. In this case, s.c. dosing of VDZ was 108 mg every 2 weeks, as per the medication manufacturer’s label. Inclusion was based on both the physician’s and patient’s intentions, while the baseline was defined as the day of the first s.c. injection, with w52 set as the follow-up. In addition, the patients without histological verification of the disease and IBD-U were excluded. The reporting of this study conforms to the STROBE statement.^26^ All of the patients provided their written informed consent prior to participation.

### Data collection

Demographic and clinical data, including sex, current age, age at diagnosis, disease duration, disease type, disease localization and extension, and behavior data (using Montreal classification^27^) were gathered at inclusion. In addition, clinical and biochemical activities were recorded, while blood samples were obtained at the baseline before the first VDZ injection and at w52 for routine laboratory testing (including C-reactive protein (CRP)) and VDZ drug-level measurement. The reason for withdrawal and further treatment were classified based on the physician’s discretion.

Secondary loss of response (LOR) was classified if a switch to another drug was necessary, due to disease activity. Injection site reactions, systemic allergic reactions, and other causes were also registered. As for clinical activity, it was defined by the partial Mayo score in UC (pMayo >2^28^) and the Harvey-Bradshaw Index in CD (HBI >4^29^), according to the standard of IBD care. Biochemical activity was objectized using CRP (>5 mg/L).^30^ Routine laboratory testing was performed immediately after sampling, while the serum samples were collected into a native blood collection tube. After coagulation and centrifugation (at 2500 rpm and 10 min), the serum samples were transferred into an 8 mL cryogenic tube and stored at −80°C until measurement. The serum VDZ levels were measured via the ELISA method (RidaScreen VDZ^®^; R-Biopharm, Pfungstadt, Germany) in 12 weeks after sample collection.

In this study, data collection was performed at regular intervals, while the participants’ data were collected into a uniform database. The patients who completed the 12-week follow-up were asked to complete a non-validated questionnaire regarding their satisfaction with the type of administration and treatment, the difficulty of s.c. administration, the reason for the therapy choice, and any adverse events and experiments.

### Assessment of the psychological components of treatment failure

An online questionnaire was completed by the patients who accepted a psychological evaluation at w52 (after switching) to explore the factors that may have influenced the treatment failure. The questionnaire included the following four psychological scales, in addition to the socio-demographic data:

#### Multidimensional Health Locus of Control

Form C of the Multidimensional Health Locus of Control (MHLC-C) measured the health control beliefs of the patients living with chronic illness. In the Hungarian version, three factors were identified: *Internal, Chance*, and *Powerful Others*.^31,32^

#### Brief Illness Perception Questionnaire

This questionnaire assessed the cognitive and emotional representations of the disease. The Hungarian version identified eight dimensions: *Consequences, Temporality, Personal Control, Treatment Control, Identity, Coherence, Concern*, and *Emotional Representation*. Question 9 asked about the patient’s perceptions of their illness. In this case, a higher score indicates a more negative perception of the illness.^33–35^

#### Inflammatory Bowel Disease Self-Efficacy Scale

This scale measured the self-efficacy of the patients using four factors based on a 10-point Likert scale: *Managing Stress and Emotions; Managing Healthcare; Managing Symptoms and Illness*; and *Being Symptom-free*.^36,37^

#### International Personality Item Pool

This 50-item questionnaire measured the five most measured personality factors, including *Extraversion, Agreeableness, Conscientiousness, Emotional Stability—Neuroticism*, and *Intellect/Openness*. In this case, each subscale included a maximum score of 50 points.^38^

### Outcome measurements and definitions

In this study, the primary outcome was the drug persistence rate at w52, while the secondary co-outcomes were the changes in the clinical corticosteroid-free remission (CSFR) and biochemical remission (BR; CRP ⩽ 5 mg/L) rates, safety issues, serum drug levels, patients’ preferences and satisfaction after switching treatment, and psychological features.

### Statistical analysis

A sample size calculation was not performed since we analyzed all of the eligible patients and there was no control group. Descriptive statistics were expressed as the mean and standard deviation or the median and interquartile range (IQR) for the continuous variables and the number and percentages for the categorical variables. Normality was tested using visual interpretations (histograms and quantile–quantile plots). After checking the assumptions, the groups with the categorical variables were compared using the chi-square test or Fisher’s exact test, while the continuous variables were compared using *t*-tests. In addition, the Kaplan–Meier survival curves with the log-rank test were used to determine the drug survival rates.

As for the effectiveness outcomes, an analysis was performed on the patients with available data, while Bonferroni correction was used to reduce the bias of multiple comparisons. A *p*-value of <0.05 indicated statistically significant differences. Statistical analysis was performed using Statistical Package for the Social Sciences (SPSS) software (IBM SPSS Statistics, version 29.0; IBM Corp., Armonk, NY) and Jamovi Statistical Software (v.1.6.9).^39^ The responses about feelings and experiences during the process were only obtained through post hoc data collection. After analyzing the socio-demographic data, the impact of treatment failure was examined using the Mann–Whitney test. Moreover, psychological factors were analyzed according to gender and disease type.

### Ethical approval

This study was approved by the National Institute of Pharmacy and Nutrition according to the Scientific Research Ethics Committee of the Hungarian Medical Research Council’s proposal (Registration No. OGYÉI/49083-1/2021) and by the Research Ethics Board at the McGill University Health Center (Approval no.: 2023-9296). This study was also conducted according to the principles of the Declaration of Helsinki. The patients in this study gave their written informed consent prior to their participation.

## Results

### Patient characteristics

In total, 70 IBD participants (32 CD and 38 UC patients) were enrolled in our study in which the male ratio was 50% and 34.2%, respectively. As for the median age at inclusion, it was 48.0 years (IQR 37–58) and 40.6 years (IQR 35–50; *p* = 0.09) in the CD and UC cohorts, respectively. In addition, the median follow-up period was 52 weeks.

Approximately half of the CD patients had ileocolonic localization (43.8%), while more than half of the UC patients had pancolitis (57.9%) at inclusion. Inflammatory behavior was registered in 59.4% of the CD cases, while prior bowel resection was more common among the CD patients (46.9% vs 5.3%, *p* < 0.001). The median i.v. VDZ treatment duration was 6.8 months (IQR 3–24) and 8.9 months (IQR 4–18) in the CD and UC cohorts, respectively. Approximately three-quarters of the patients were in CSFR at the baseline, while the CRP showed mild elevation in both cohorts (CD 6.0 (± 5.3); UC 6.1 (± 6.9)). In addition, oral 5-ASA was the most common concomitant medication at inclusion in both diseases (oral 5-ASA: CD 25.0% and UC 47.4%). Further details of the patient’s clinical and demographic data are presented in Table 1.

**Table 1.** Baseline demographic and clinical characteristics of enrolled patients.

Variables	Total (*n* = 70)	CD (*n* = 32)	UC (*n* = 38)	Sig.
Follow-up duration, weeks, median (IQR)	52.0 (52–52)	52.0 (52–52)	52.0 (52–52)	0.221
Sex, male (%)	29 (41.4)	16 (50)	13 (34.2)	0.137
Age at inclusion, years, median (IQR)	43.2 (36–57)	48.0 (37–58)	40.6 (35–50)	0.094
Disease duration at inclusion, weeks, median (IQR)	11.0 (9–17)	12.0 (9–23)	10.5 (8–15)	0.154
Prior bowel resection, *n* (%)	17 (24.3)	15 (46.9)	2 (5.3)	<0.001
IV VDZ treatment duration, weeks, median (IQR)	8.0 (4–20)	6.8 (3–24)	8.9 (4–18)	0.994
Disease extent, *n* (%)^a^				
Proctitis	5 (7.1)		5 (13.2)	
Left-sided	11 (15.7)		11 (28.9)	
Pancolitis	22 (31.4)		22 (57.9)	
Disease localization, *n* (%)^a^				
Ileal	7 (10.0)	7 (21.9)		
Colonic	11 (15.7)	11 (34.4)		
Ileocolonic	14 (20.0)	14 (43.8)		
Upper GI disease	1 (1.4)	1 (3.1)		
Disease behavior, *n* (%)^a^				
Inflammatory	19 (27.1)	19 (59.4)		
Stricturing	6 (8.6)	6 (18.8)		
Penetrating	7 (10.0)	7 (21.9)		
Perianal disease	6 (8.6)	6 (18.8)		
Disease activity				
pMayo, mean (±SD)			1.3 (1.8)	
HBI, mean (±SD)		2.3 (2.1)		
CSFR, *n* (%)	53 (75.7)	26 (81.3)	27 (71.1)	0.322
CRP, mg/L, mean (±SD)	6.1 (6.2)	6.0 (5.3)	6.1 (6.9)	0.949
Concomitant treatment at baseline, *n* (%)				
Oral 5-ASA	26 (37.1)	8 (25.0)	18 (47.4)	
Immunomodulator	8 (11.4)	2 (6.3)	6 (15.8)	
Systemic corticosteroid	7 (10.0)	3 (9.4)	4 (10.5)	

a Montreal classification.

CD, Crohn’s disease; CRP, C-reactive protein; CSFR, corticosteroid-free clinical remission; GI, gastrointestinal; HBI, Harvey-Bradshaw Index; IQR, interquartile range; IV, intravenous; *n*, number of patients; pMayo, partial Mayo score; UC, ulcerative colitis; VDZ, vedolizumab.

### Treatment persistence

Overall, 17.1% of the patients (12/70) stopped s.c. VDZ treatment by the follow-up, after a median of 26.2 weeks (IQR 20–47). Specifically, they included 21.9% (7/32) in the CD cohort and 13.2% (5/38) in the UC cohort (*p* = 0.34), after a median of 26.4 weeks (IQR 1–50) and 26 weeks (IQR 6–47; *p* = 0.296), respectively.

**Figure 1.**
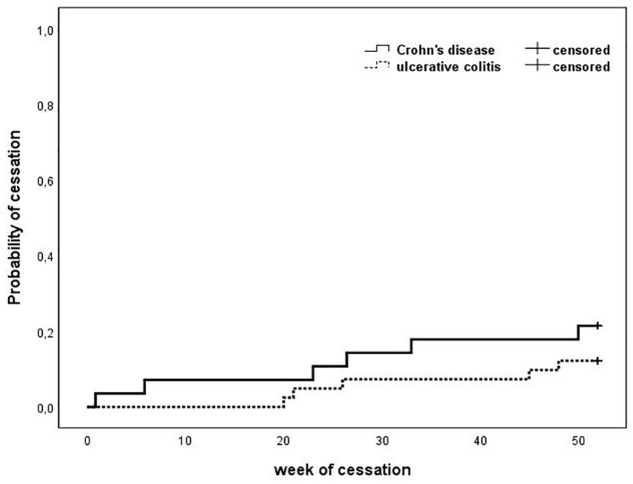
Kaplan–Meier survival characteristics of the patients. The solid line represents the CD patients, while the dashed line represents the UC patients (*p* = 0.255). CD, Crohn’s disease; UC, ulcerative colitis.

In the CD group, 2/7 patients switched to ustekinumab (CRP = 32 mg/mL and HBI = 7) and infliximab (CRP = 18 mg/mL and HBI = 5). In the UC group, LOR was registered in 1/5 of patients who switched to tofacitinib (CRP = 2.5 mg/mL and pMayo = 6). In the case of two CD patients and one UC patient, s.c. treatment was terminated because of local injection site reactions (erythema, induration), after which they switched back to i.v. formulation. Meanwhile, one CD patient with a fear of needles continued treatment on the i.v. regimen. The follow-up was concluded after 1/7 CD patients and 3/5 UC patients requested to switch back to i.v. administration, without any sign of disease aggravation. Finally, one CD patient terminated the VDZ treatment, due to chemotherapy of a novel colonic cancer. The survival characteristics of the cohorts are presented in Figure 1.

### Treatment effectiveness and therapeutic drug levels

Overall, 53/70 (75.7%) patients were in CSFR at the baseline, with 26/32 (81.3%) patients in the CD cohort and 27/38 (71.1%) patients in the UC cohort. The CSFR rates did not significantly change among the CD patients (23/25, *p* = 0.25), whereas all of the UC patients (33/33, *p* < 0.001) went into remission by w52.

**Figure 2.**
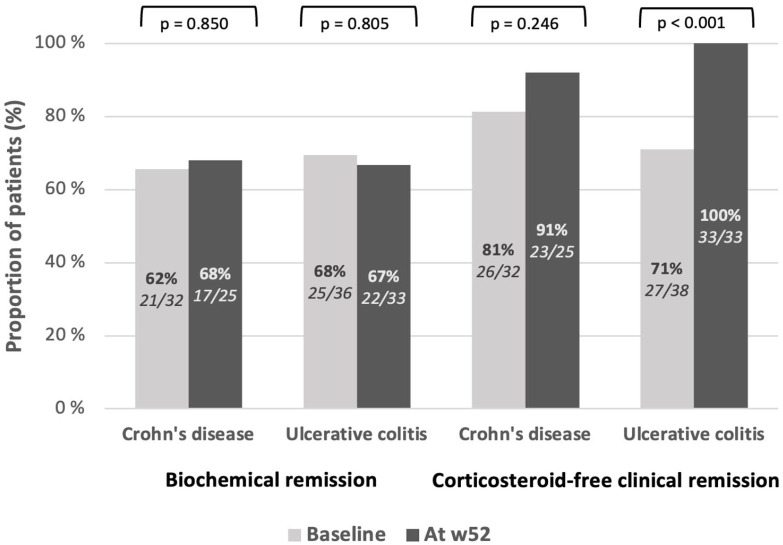
The proportion of patients achieving biochemical (CRP < 5 mg/mL) and corticosteroid-free clinical (HBI <5, pMayo <3) remission at the baseline and by week 52. CRP, C-reactive protein; HBI, Harvey-Bradshaw Index; w52, week 52; *p*, significance level.

The BR rate was 46/70 (65.7%) in the total cohort, with 21/32 (65.6%) patients in the CD cohort and 25/36 (69.4%) patients in the UC cohort. The BR rates did not change among the CD (17/25, *p* = 0.85) or UC (22/33, *p* = 0.81) groups by w52. The clinical and BR rates are presented in Figure 2.

**Figure 3.**
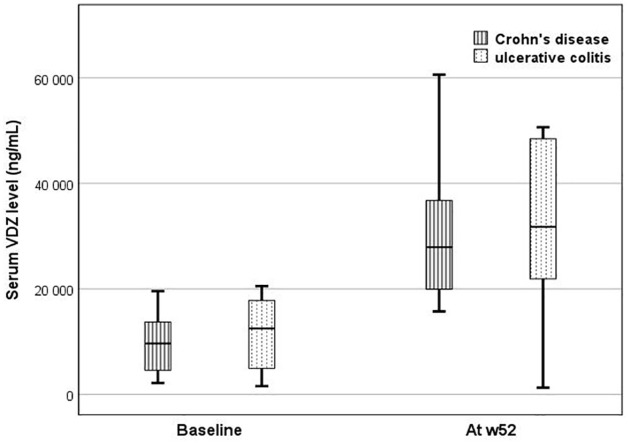
Serum VDZ levels of the patients by week 52. In both the CD and UC groups, the serum VDZ levels increased by w52 (*p* = 0.047 and *p* = 0.001). CD, Crohn’s disease; UC, ulcerative colitis; VDZ, vedolizumab; w52, week 52.

The serum VDZ levels were measured at the baseline and w52. Overall, the serum VDZ levels increased by w52 from a mean of 11.8 (± 12.4) µg/mL to 31.2 (± 17.2) µg/mL (*p* < 0.001). Specifically, in the CD patients, the levels increased from a mean of 8.9 (± 6.9) µg/mL to 30.6 (±17.8) µg/mL (*p* = 0.047), while in the UC patients, the levels increased from 13.2 (± 14.5) µg/mL to 31.6 (± 17.8) µg/mL (*p* = 0.003). The serum VDZ levels did not differ at the baseline (CD 8.9 ± 6.9 µg/mL and UC 13.2 ± 14.5 µg/mL; *p* = 0.26) or by w52 (CD 30.6 ± 17.8 and UC 31.6 ± 17.8 µg/mL, *p* = 0.95). The serum VDZ levels are presented in Figure 3.

### Patient satisfaction

Overall, 28 patients (CD = 11) completed the questionnaire regarding treatment satisfaction. Specifically, the decision on treatment change was affected by the physician’s advice in 22 (78.6%) cases, the possibility of home application in 7 (7.1%) cases, and time-sparing in 7 (7.1%) cases. Meanwhile, subjective effectiveness was high on a 1–3 scale (median = 1 (IQR 1–2)), with no difference according to disease type. The majority of the patients (96.4%) administered the s.c. VDZ injections themselves, indicating that such injections were not difficult (2 (IQR 1–2), based on a 1–10 scale). Among the switched patients, 78.6% administered the injections themselves at home.

### Psychological factors affecting treatment survival

Among the participants who accepted psychological analysis (37/70), 54.1% were CD patients (male/female ratio was 45.9%; median age was 43.9 years (IQR 35–50)). Overall, 29.7% of the patients discontinued treatment and 27% switched back to i.v. treatment. In addition, 72.9% of the patients had a high school degree or higher, 86.5% were in a relationship or married, and 86.5% had not been treated for any psychiatric problems in the past 3 years. Interestingly, 86% of the respondents assumed that some psychological factor plays a role in their bowel disease. Specifically, 16% only cited psychological factors as the cause of their illness, 45.9% cited psychological and lifestyle factors, and 24.3% suspected psychological, genetic, and lifestyle factors as the cause of their illness. Further details of the variables are as follows.

#### Health control beliefs

There was no significant difference between those who switched back to i.v. treatment and those who did not (*Internal: p* = 0.22; *Chance: p* = 0.61 *Powerful Others: p* = 0.74). However, gender showed a significant difference in terms of the *Chance* subscale since the males showed a higher proportion of external control attitudes (*p* = 0.009). Meanwhile, there was no significant difference in the health locus of control by disease type (*Internal: p* = 0.33; *Chance: p* = 0.70, *Powerful Others: p* = 0.84).

#### Illness perception

No significant difference in illness perception was found between those who switched back to i.v. treatment and those who did not (*p* = 0.93). However, a significant difference was found in the temporality dimension of illness perception since the females perceived a longer disease course than the males (*p* = 0.015). The dimension of coherence also showed a significant difference, with the males perceiving their disease as more coherent (*p* = 0.027). Meanwhile, the other dimensions did not show significant differences, including disease type, which had no impact on the overall score of illness perception (*p* = 0.90).

#### Disease-specific self-efficacy

No statistically significant difference in disease-specific self-efficacy was found between those who switched to i.v. treatment and those who did not (*p* = 0.72). In addition, there was no significant gender difference in self-efficacy (*p* = 0.29) and no significant difference in disease-specific self-efficacy based on disease type (*p* = 0.44).

#### Personality factors

No statistically significant difference in the personality factors was found between those who switched to i.v. treatment and those who did not. Moreover, the female patients had significantly higher medians for *Agreeableness* (*p* = 0.004), *Neuroticism* (*p* = 0.044), and *Openness* (*p* = 0.026), compared to the male patients. Meanwhile, there was no significant difference in the personality factors between the CD and UC patients.

## Discussion

In this study, the real-world experience of switching to s.c. VDZ treatment for IBD patients was assessed according to a multicenter cohort, including Hungarian and Canadian IBD centers. Although the drug survival rate was high (82.9%), a relatively high number of participants requested to switch back to i.v. administration, despite the low LOR rate at w52 and the fact that no substantive safety concerns were raised. In addition, the clinical and BR rates were stable, while the serum VDZ levels increased by w52.

Several observational studies have reported the effectiveness and safety of s.c. VDZ treatment after switching from i.v. treatment. However, their study designs and follow-up durations differed, with limited patient perspectives and a lack of psychological factors associated with treatment failure.

Treatment persistence after 1 year of switching was analyzed as the primary endpoint in our cohort, indicating treatment success in 82.9% of the participants, with no significant difference according to the disease cohorts. In the largest cohort of IBD patients in a controlled trial in the United Kingdom, similar persistence rates were reported on s.c. versus i.v. treatment (81.1% vs 81.2%).^40^ In the study by Volkers et al., 88.1% were on s.c. treatment at 1 year, while 4/11 CD patients experienced LOR and switched to another drug.^20^ In a Croatian study, LOR was observed in 16.5% of the IBD patients in the 6-month follow-up, although the escalated regimen was also included in the endpoint.^18^ In a German cohort of IBD patients by Kubesch et al., they reported a 12.2% failure rate within 20 weeks, after which half of the patients switched back to the i.v. regimen and five patients switched to other biological regimens.^22^ A similar rate was observed by Bergquist et al. at 1 year (88.5%).^21^ In the largest cohort of UC patients by Ribaldone et al., they reported an 88.7% treatment persistence rate, while 10/19 patients switched back to i.v. administration due to drug failure, adverse events, or patient’s choice.^16^ In another Italian study based on 24 months of data, they found higher failure rates among CD patients.^24^ In our study, the factors associated with LOR were not analyzed, whereas only 3/12 cases were registered as LOR. Although this made it impossible to compare the failure and success cohorts, it emphasized the high effectiveness of s.c. VDZ after switching. Furthermore, several publications reported the effectiveness of treatment based on clinical and/or BR rates^17–19,20,23^ and found stable remission rates during the follow-up, which is in line with our data.

Nevertheless, in our cohort, 41.7% of the patients decided to switch back to i.v. formulation, even with the absence of any sign of disease activity. This indicates that there was no significant difference between treatment discontinuation or return to i.v. treatment for any of the psychological factors examined. Moreover, there were no significant differences by disease type for any of the psychological factors. However, we found several differences by gender since the female IBD patients perceived a longer disease course and showed more neurotic traits than the male IBD patients. The males also perceived their illness as more coherent than the females and were more likely to have an external control attitude. Thus, they were more likely to think that luck or fate was influencing their condition.

Although exact psychological factors associated with re-switching to i.v. formulation without disease activity and valid LOR were unavailable, a French cohort study assessed the acceptance for switching from i.v. infliximab or VDZ to s.c. and found an association between refusal and treatment duration of the i.v. regimen. This may explain our low number of enrolled patients since the median i.v. treatment duration was 8 years in our cohort. Furthermore, the fear of LOR, increased frequency of administration, and self-administration may have impacted the decision-making of the patients who refused to switch.^41^ However, related research showed that switching back to i.v. is safe, as shown in a case of generalized urticaria and angioedema during s.c. admission.^42^

Meanwhile, the safety characteristics of s.c. VDZ have been reported by several papers. The most common adverse events were injection site reactions in 3% to 20% of the patients^17,20,23,40^ while infections (COVID-19, urinary tract, and gastrointestinal tract) were registered in several cases.^18,20,24^ In our study, injection site reactions occurred in 4.3% of the patients, with VDZ administration terminated in one patient, due to the treatment of a novel colonic cancer. Several cases of neoplasms were also reported in other publications, although the exact pathological connection cannot be assumed.^16,26^

Although our study includes several strengths, two limitations should be noted. First, despite the multicenter setting and relatively long follow-up period, we were unable to recruit more participants. This may be due to the low willingness of the patients to switch to s.c. formulation after undergoing VDZ treatment for such a long time. This low number also made it impossible to draw exact conclusions. Second, a high number of patients did not complete the questionnaire on satisfaction, while approximately half of the patients did not accept a psychological evaluation, which clearly limited our interpretation. However, our study adds to the literature on patients’ preferences, especially in regard to central European and Canadian experiences.

## Conclusion

In conclusion, the transition from i.v. to s.c. VDZ treatment was effective in this study, the overall persistence rate was associated with high serum drug levels, and no novel safety issues were reported. Interestingly, although s.c. administration after induction can save resources, some patients still insisted on i.v. VDZ treatment, due to its proven formulation.

## Declarations

**Ethics approval and consent to participate:** The study was approved by the National Institute of Pharmacy and Nutrition according to the Scientific Research Ethics Committee of the Hungarian Medical Research Council’s proposal (Registration no. OGYÉI/49083-1/2021) and by the Research Ethics Board, McGill University Health Centre (Approval no.: 2023-9296). The study was conducted according to the principles of the Declaration of Helsinki. Patients included in the prospective study have given written informed consent for regular healthcare and participation.

**Consent for publication:** T.M. and P.L.L. is the guarantor of the article. All authors have read and agreed to the submitted version of the article.

**Availability of data and materials:** P.L.L. and T.M. were the guarantors of this manuscript. All of the authors agreed to the submitted version of the manuscript. Although the data are available upon request, they cannot be shared publicly due to the privacy of the participants. This manuscript, including the related data and figures, has not been previously published and is not under consideration elsewhere.
